# Genome-resolved transcriptomics reveals novel PCE-dehalogenating bacteria from Aarhus Bay sediments

**DOI:** 10.1128/msystems.01503-24

**Published:** 2025-04-16

**Authors:** Chen Zhang, Tom N. P. Bosma, Siavash Atashgahi, Hauke Smidt

**Affiliations:** 1 Laboratory of Microbiology, Wageningen University and Research 568404, Wageningen, the Netherlands; University of Massachusetts Amherst, Amherst, Massachusetts, USA

**Keywords:** organohalide respiration, metagenomics, metatranscriptomics, RDase gene, *Vulcanibacillus*, transcriptional regulators

## Abstract

Organohalide-respiring bacteria (OHRB) are keystone microbes in bioremediation of sites contaminated with organohalides and in natural halogen cycling. Known OHRB belong to distinct genera within the phyla *Chloroflexota*, *Proteobacteria*, and *Firmicutes*, whereas information about novel OHRB mediating natural halogen cycling remains scarce. In this study, we applied a genome-resolved transcriptomic approach to characterize the identity and activity of OHRB from tetrachloroethene respiring cultures previously enriched from sediments of Aarhus Bay. Combining short- and long-read sequencing approaches, we assembled 37 medium-quality bins with over 75% completeness and less than 5% contamination. Sixteen bins harbored RDase genes and were affiliated taxonomically to the class of *Bacilli* and phyla of *Bacteroidota*, *Synergistota*, and *Spirochaetota*, which have not been reported to catalyze reductive dehalogenation. Among the 16 bins, bin.26, phylogenetically close to the genus *Vulcanibacillus* (phylum *Firmicutes*), contained an unprecedented 97 reductive dehalogenase (RDase) genes. Of these, 84 RDase genes of bin.26 were transcribed during tetrachloroethene dechlorination in addition to RDase genes from the members of *Synergistales* (bin.5 and bin.32) and *Bacteroidales* (bin.18 and bin.24). Moreover, metatranscriptome analysis suggested that the RDase genes were likely under the regulation of transcriptional regulators not previously associated with organohalide respiration, such as HrcA and SigW, which are known to respond to abiotic environmental stresses, such as temperature changes. Combined application of genomic methods enabled us to pinpoint novel OHRB from pristine environments not previously known to mediate reductive dechlorination and to add to the current knowledge of the diversity, activity, and regulation of RDase genes.

**IMPORTANCE:**

Pristine marine environment is the major reservoir for naturally produced organohalides, in which reductive dehalogenation underneath plays an important role in the overall cycling of these compounds. Here, we obtain some novel OHRB genomes from Aarhus Bay marine sediments, which are phylogenetically distant to the well-documented OHRB and widely distributed across the bacterial phyla, such as *Bacteroidota*, *Synergistota*, and *Spirochaetota*. Furthermore, transcriptional profiles unravel that these RDase genes are induced differently, and their activity is controlled by diverse regulatory systems. Accordingly, elucidating the reductive dehalogenation of pristine marine environments substantially advances our understanding of the diversity, phylogeny, and regulatory variety of dehalogenating bacteria contributing to the global halogen cycle.

## INTRODUCTION

Organohalide respiring bacteria (OHRB) can derive energy for growth from the use of reductive dehalogenation of halogenated compounds as a terminal electron accepting process. They employ reductive dehalogenases (RDases) that catalyze the removal of halide(s) from the carbon backbone via respiratory electron transfer (1, 2). In the process of organohalide respiration (OHR), organohalides serve as the terminal electron acceptor for the electrons derived from, for example, hydrogen or lactate, through a membrane-associated electron transport chain (ETC) (3–5). In addition to RDases known from OHRB, reductive dehalogenation can also be catalyzed by another type of reductive dehalogenase, thiolytic, glutathione-dependent tetrachloro-P-hydroquinone RDase (TPh-RDase) as previously reported for the biodegradation of chlorinated compounds (6, 7). In many cases, RDase gene-containing gene clusters in OHRB are composed of genes encoding a transcriptional regulator, the RDase itself, and a potential electron transporter. Thereinto, three regulatory systems of RDase gene clusters have been characterized: CRP (cyclic AMP receptor protein)/FNR (fumarate and nitrate reduction regulator) superfamily, two-component systems (TCS), and MarR (multiple antibiotic resistance regulator) (8, 9). The minimum cassette of RDase gene clusters is usually composed of a gene encoding the catalytic subunit, *rdhA*, and a second gene, *rdhB,* coding for a cognate membrane-anchoring protein B (3, 4, 10). Organohalides are present as natural products in marine environments and other high salt environments, such as tetrachloroethene (PCE) that can be produced by algae (11–13), which is why it is interesting to study their role in the geochemical carbon and halogen cycles and their potential eco-physiological importance.

OHR exploration in marine sediments has been a long-term topic for several decades, which was mainly based on culture-dependent and molecular detection methods, such as dilution-to-extinction isolation, marker-gene amplicon sequencing, and quantification (14–16). Recent research into the microbial composition of marine sediments, using metagenomic techniques and bioinformatics, has revealed the presence of microorganisms from various phyla containing genes predicted to code for RDases, including, next to the commonly known phyla, such as *Chloroflexota* and *Firmicutes*, new archaeal phyla, *Lokiarchaeota*, *Thorarchaeota*, and *Heimdallarchaeota* belonging to the proposed *Asgardarchaeota* superphylum (17).

Based on single-cell amplified genomes (SAGs) retrieved from the sediment of Aarhus Bay, a putative RDase gene was found in the assembled genome DEH-C3, belonging to the *Dehalococcoidia* (18). Metatranscriptomic data from the surroundings of the initial sampling site revealed a high abundance of *tceA*-like gene transcripts implicating the potential for reductive dehalogenation of PCE or trichloroethene (TCE) in this marine sediment from Aarhus Bay (19). Moreover, five *Desulfatiglans*-related SAGs were found bearing putative RDase genes from the sulfate-rich subsurface of Aarhus Bay marine sediments, and it was speculated that OHR might be an alternative energy conservation strategy under sulfate-limiting conditions (20, 21). Considering the potential inhibitory effect of sulfate reduction on reductive dehalogenation caused by the produced and accumulated sulfide (22, 23), the presence or absence of sulfate was taken into account for OHR exploration in Aarhus Bay marine sediments. Moreover, we previously showed that sulfate reduction significantly shaped the PCE-dechlorinating community compared with corresponding incubations without additional sulfate (24). Nevertheless, although these analyses of extensive DNA and RNA sequence data indicated the existence of potential OHR in marine sediments of Aarhus Bay, unambiguous physiological proof remains pending, and the OHRB involved were not isolated nor unequivocally identified.

In a previous study, we found that marine sediments from Aarhus Bay can dehalogenate a range of halogenated compounds, including PCE, 2,6-dibromophenol (2,6-DBP), 1,4-dibromobenzene (1,4-DBB), 3-bromophenol (3 BP), and 2,4,6-triiodophenol (2,4,6-TIP) (24). 16S ribosomal RNA (rRNA) gene amplicon sequencing revealed that members of the phyla *Desulfobacterota*, *Firmicutes,* and *Bacteroidota*, such as *Desulfovibrio*, *Desulfuromusa,* and *Bacillus*, were enriched in sediment-free PCE dechlorinating cultures. *Desulfovibrio* and *Desulfuromusa* were recently shown to be capable of reductive debromination of 2,6-DBP to phenol (25). However, *Bacillus* spp. or members of the *Bacteroidota* have never been reported before to use organohalides as terminal electron acceptors.

Thus, the aim of this study was to identify the OHRB involved in the dehalogenation of PCE aligning with the previously reported study (24). To this end, we combined metagenomics and transcriptomics of sediment-free PCE-dechlorinating enrichment cultures with physiological observations in order to identify the corresponding OHRB and their RDase genes for PCE dechlorination with(out) additional sulfate. This revealed the presence of a large number of genomic bins encoding putative reductive dehalogenases, as well as the expression of many of these genes that appeared to be under the control of transcriptional regulators not previously associated with OHR.

## MATERIALS AND METHODS

### Chemicals

PCE, TCE, cis-dichloroethene (cDCE), vinyl chloride (VC), and ethene were purchased from Sigma-Aldrich. Stock solutions of lactate (0.5 M) and sulfate (0.5 M) were prepared separately by filter sterilization (syringe filter, 0.2 µm, mdimembrane, Ambala Cantt, India). All other (in)organic chemicals were obtained in analytical grade or higher.

### Cultivation

Cultures were incubated in the marine medium under sulfate-free (NS) or sulfate-amended (S) conditions as described previously (24). These cultures labeled as PCE_NS_Tr2_A/B (PCE.NS.Tr2.A/B) and PCE_S_Tr2_A/B (PCE.S.Tr2.A/B) (24), grown in 50 mL per bottle, were the same cultures as indicated in step 4 in the experimental outline of previous work (24). Briefly, the cultures were obtained after three spikes (see below) that were applied when PCE was completely dechlorinated into cDCE. Each spike contained 250 µM PCE and 5 mM lactate (NS and S), and 5 mM sulfate in S cultures. The actively dechlorinating cultures, PCE_NS_Tr3 and PCE_S_Tr3 (50 mL per bottle), respectively, were obtained after 5% transfer of PCE_NS_Tr2_A and PCE_S_Tr2_A. The remainders of PCE_NS_Tr2_A and PCE_S_Tr2_A were sacrificed for metagenome sequencing by Illumina (Novogene Europe, Cambridge, UK). PCE_NS_Tr2_B and PCE_S_Tr2_B cultures were used for metagenome sequencing by Nanopore separately (Novogene Europe). When 80% of the last of three spikes of 250 µM PCE was consumed, PCE_NS_Tr3 and PCE_S_Tr3 were transferred at 5% in volume to PCE_NS_Tr4 and PCE_S_Tr4, respectively, in three replicates (100 mL per bottle). Similar to the cultures used for metagenome sequencing, three replicate cultures of PCE_NS_Tr4 and PCE_S_Tr4 were collected for metatranscriptome sequencing by Illumina (Novogene Europe) when 80% of the PCE was dechlorinated into cDCE after the third spike. PCE dechlorination was not synchronized under NS and S conditions, causing the harvest of the respective cultures at different time points.

### Analytical methods

PCE, TCE, and cDCE were measured by gas chromatography and mass spectroscopy (GC-MS) installed with an Rt-Q-BOND column (Retek, PA, USA) and a DSQ MS (Thermo Fisher Scientific). Hydrogen and methane were detected by compact GC (Global Analyzer Solutions, Breda, The Netherlands) with a thermal conductivity detector (GC-PDD). Organic acids, including lactate, acetate, and propionate, were measured using SHIMADZU LC2030 PLUS coupled with a Shodex SUGAR Series SH1821 column. Sulfate was measured using the Dionex ICS-2100 Ion Chromatography System (Thermo Scientific), and sulfide was analyzed photometrically as previously described (26).

### DNA and RNA extraction

Cultures used for DNA and RNA extraction were centrifuged at 10,000 × *g* for 5 min and then washed three times with 10 mM TE buffer (pH 7.0) to remove residual medium components. Washing of cultures for RNA extraction was done at 4°C. DNA extraction was done by using the MasterPure gram-positive DNA purification Kit (Epicentre, WI, USA) to gain high-quality and quantity DNA for metagenomic sequencing, including Illumina for short reads (PE 150, NovaSeq 6000) and Oxford Nanopore for long reads. The RNA extraction was carried out following a bead-beating procedure (27). The isolated RNA was purified using RNeasy columns (Qiagen, Venlo, The Netherlands), and residual genomic DNA was subsequently digested by DNase I (Roche, Almere, The Netherlands). The obtained RNA samples were quality-checked by agarose gel electrophoresis and sequenced using Illumina for short reads (PE 150, NovaSeq 6000).

### Metagenome data analyses

Metagenome sequence data were generated by Illumina sequencing in paired-end short reads (NovaSeq 6000) and Oxford Nanopore (PromethION). The pipelines of MetaWRAP (v1.3), OPERA-MS (v0.8.3), and Anvi’o (v7.1) were combined to process the raw data as outlined in the following (Fig. S1) (28–30). One of the duplicate cultures, PCE_NS_Tr2_A and PCE_S_Tr2_A, was sent for paired-end Illumina sequencing. Cleaning of short reads was achieved through quality check and trimming using the “read_qc” module of MetaWRAP. Both forward and reverse sequencing of PCE_NS_Tr2_A yielded 33,352,593 reads, respectively, with 33,340,280 read pairs remaining after filtering for human genomic DNA contaminants and quality control. Raw and filtered read pairs of PCE_S_Tr2_A amounted to 26,516,123 and 26,506,140, respectively. Co-assembly of the clean reads from both cultures was done using the “assembly” module of MetaWRAP (metaSPAdes-3.10 and MEGAHIT-1.2.9). PCE_NS_Tr2_B and PCE_S_Tr2_B cultures were used for Nanopore sequencing. The number of filtered reads of PCE_NS_Tr2_B and PCE_S_Tr2_B was 1,637,484 and 954,603, respectively. The short-read co-assembly was then combined with the Nanopore long reads using OPERA-MS (Fig. S1). Subsequently, metatranscriptome sequences (see below for processing details) were introduced to improve the quality of the hybrid assembly by OPERA-MS (Table S1). As a next step, the hybrid assembly was used for binning using the “binning” module of MetaWRAP (v1.2.3) separately with three commonly used built-in binners: metabat2, maxbin2, and concoct. Further refining of bins was achieved using the “bin_refinement” module and selection of bins using a cutoff at 50% completeness and 10% contamination. The abundance of the refined bins was determined using the “quant_bins” module in MetaWRAP, which uses Salmon to index the entire metagenomic assembly, and then, reads from samples were mapped back to the hybrid assembly. The generated coverage estimates were used to calculate the abundance of each contig in each sample. Length-weighted average of a given bin’s contig abundances was used to calculate the bins’ abundances. Bin quality was further improved using the “reassemble_bins” module of MetaWRAP. The reassembled bins were then classified using the “classify_wf” module of GTDB-Tk (v2.0.0) (31), and phylogenomic analysis of bins was performed on the Anvi’o platform annotated with the “annotate_bins” module of MetaWRAP using the built-in PROKKA (v1.14.5). To assess the genomic context of target genes, flanking genes were searched by setting “grep -A 6 -B 5” to fetch the first five upstream genes and the first six downstream genes using the translated “.faa” format files. The obtained gene clusters were visualized by “gggenes” (https://github.com/wilkox/gggenes).

### Phylogenetic analysis of OHR bins and RDases

Thirty-seven bins, also termed Metagenome-Assembled Genomes (MAGs), were retained after filtering using the “Bin_refinement” and “reassemble_bins” modules of MetaWRAP, with settings of >75% completeness and <5% contamination. These 37 MAGs were then phylogenomically analyzed following the Anvi’o workflow using the “--hmm-source Bacteria_71,” and the output tree file was visualized by ggtree (32), which combined the bins’ abundance, transcripts, and their genome size. Bins of potentially reductively dehalogenating bacteria were identified using the curated HMM (Hidden Markov Model) files of RDase genes, which includes respiratory reductive dehalogenases of OHRB containing three conserved motifs, twin-arginine translocation signal peptide (TAT), and two motifs of binding iron-sulfur clusters in general, and tetrachloro-p-hydroquinone (TPh) reductive dehalogenase using “reductive_dehalogenase.hmm” with “bitscore_cutoff” value set at 132 (https://github.com/Arkadiy-Garber/MagicLamp) as described in “hmm-meta.txt” in the “hmms” directory, which had the same output by using PF13486 (6, 7, 33). To establish their evolutionary position, bins were taxonomically classified by “classify_wf” of GTDB (34), and bins from the same phylum or class were collected together with selected representative genomes from the respective phylum from the GTDB database (Using GTDB v202) to construct phylogenetic trees using “GToTree (v1.6.35)” (35) using 74 bacterial single-copy protein-coding genes (SCGs) for concatenated alignment. In addition, RDase entries from the Pfam database (PF13486, http://pfam.xfam.org/) were employed to retrieve all potential OHRB genomes related to bins at the phylum- or class-level and listed together in Table S3 (10^th^, Apr 2022). Similarly, 114 RDase protein sequences were collected from 16 bins, and 35 RDase representatives from ortholog groups (OGs), established upon 90% full-length protein pairwise identity (PID) and the functionally identified reductive dehalogenases (36–41), were added together for multiple sequence alignments using the online Clustal Omega tool to construct a phylogenetic tree (42). SIAS was employed to calculate the Sequence Identity And Similarity (http://imed.med.ucm.es/Tools/sias.html). RDase protein sequences are listed in Table S4.

### Metatranscriptome data analyses

Similar to the processing of metagenome data, raw Illumina metatranscriptome sequence data were first cleaned through the removal of human genomic contaminants that may be introduced during sample preparation and extraction. Similarly, Salmon as built in the quant_bin module of MetaWRAP (v1.1.6) was also employed to quantify the activity of bins based on transcript mapping to the respective contigs. Tuxedo packages (https://github.com/trinityrnaseq) were used for genome-guided RNA-seq analysis, including Tophat, Cufflinks, Cuffmerge, and Cuffdiff (Fig. S1). The hybrid assembly used for the binning (see above) was set as the template for aligning the RNA-seq data by Tophat. Cufflinks were used for assembling transcript structures from read alignments, and transcripts were counted based on Cuffdiff output, which was used for performing differential expression analysis. The abundance of standardized transcripts was taken into account to construct expression profiles of functional genes under NS and S conditions.

### Functional analyses of metabolic pathways encoded in the different bins

To evaluate the potential metabolism encoded in the 37 assembled bins, MagicLamp was also employed to search metabolic marker genes according to the established HMM files as described in “hmm-meta.txt” in the “hmms” directory (33). In our physiological experiments, we observed, alongside reductive dehalogenation, sulfate reduction as well as hydrogen and methane production, and relevant marker genes were selected. For sulfur metabolism, genes related to sulfide/sulfur/thiosulfate oxidation, sulfite/sulfate reduction, and thiosulfate disproportionation were retrieved. The initial step of sulfate reduction is the reduction of sulfate to sulfite with the formation of adenosine 5’-phosphosulfate (APS) as the intermediate, catalyzed by sulfate adenylyl-transferase (Sat), and adenylyl-sulfate reductase (Apr) reducing APS to sulfite (43–45). Sulfite reductase is the critical enzyme to catalyze the reduction of sulfite to sulfide, which is the limiting step for sulfate reduction (46, 47). Two types of sulfite reductase genes were taken into consideration, including those encoding dissimilatory sulfite reductase (*dsr* genes) and anaerobic sulfite reductase (*asr* genes) (48, 49). Marker genes encoding enzymes involved in the halogen cycle were selected in addition to RDase and TPh-RDase genes, including genes encoding haloacid dehalogenase type II for organohalide breakdown, DMSO reductase type II PcrA/B for perchlorate reduction, and chlorite dismutase for chlorite reduction (50–54). Marker genes coding for hydrogenases were divided into three groups according to the metal content at active sites, that is, [Ni-Fe]-hydrogenase, [Fe-Fe]-hydrogenase, and [Fe]-hydrogenase (55, 56). For the well-studied [Ni-Fe]-hydrogenases, eight subgroups, group 1, group 2a, group 2b, group 3a, group 3b, group 3c, group 3d, and group 4, and two subgroups of [Fe-Fe]-hydrogenases, group A and group B, were included (55–57). Metabolic genes responsible for methane production and oxidation were also searched against the assembled bins, which included *pmoA*, *pmoB*, *pmoC*, *mmoB*, and *mmoD* for methane oxidation, and *mcrA*, *mcrB*, and *mcrG* for methane production (58, 59).

## RESULTS

### PCE dechlorination to cDCE

**Fig 1:**
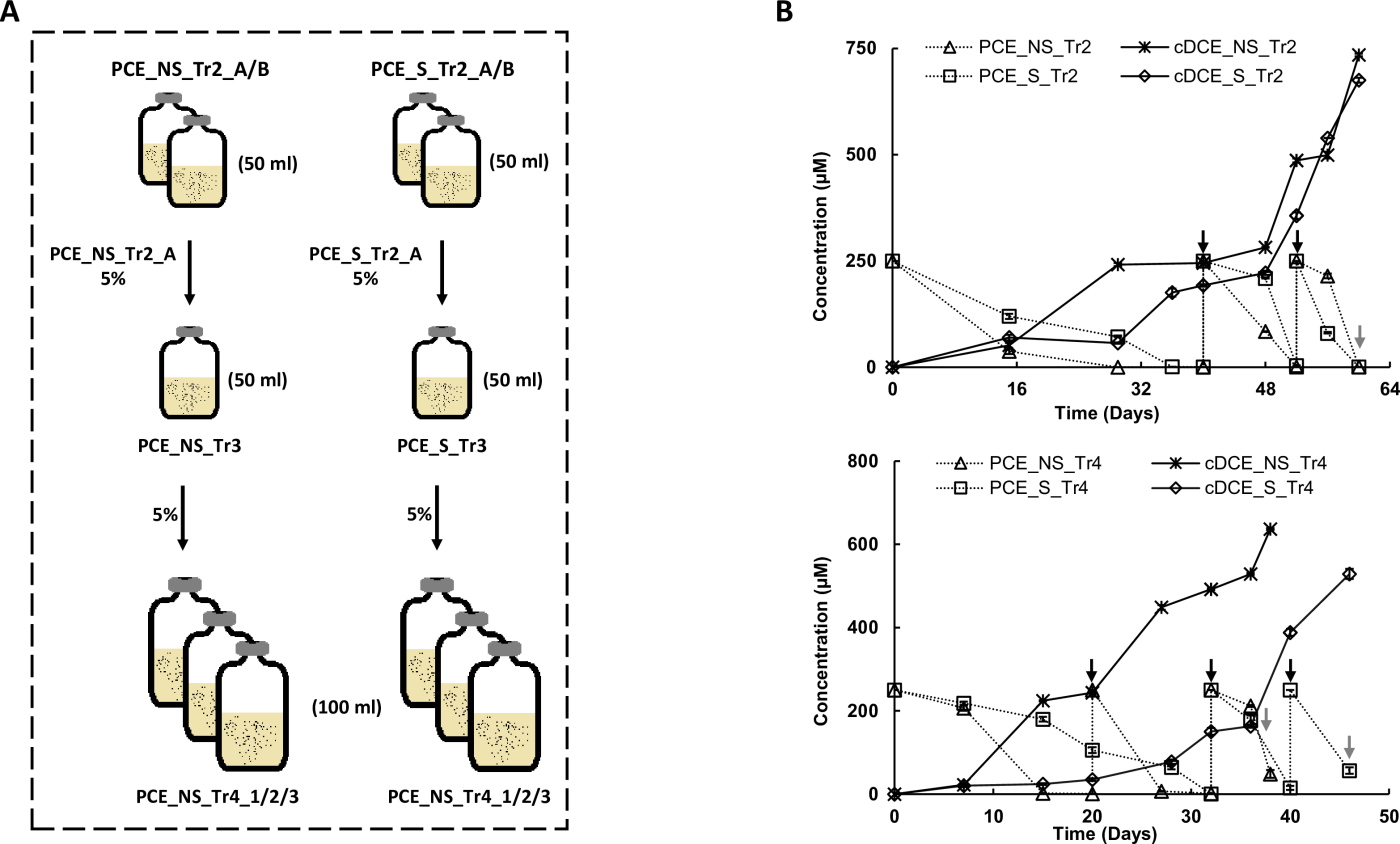
Schematic flowchart (**A**) of PCE dechlorinating cultures (**B**) sampled for meta-genome and -transcriptome sequencing; 5% denotes the transfer volumes of 5% (vol/vol) to the subsequent cultures; PCE_NS_Tr2_A/B: duplicate PCE dechlorinating sulfate-free cultures; PCE_S_Tr2_A/B: duplicate PCE dechlorinating sulfate-amended cultures; PCE_NS_Tr4_1/2/3 and PCE_S_Tr4_1/2/3: triplicate PCE dechlorinating cultures; black arrows in B indicate PCE spikes; and gray arrows represent timepoints at which cultures were harvested.

Stable, sediment-free PCE-dechlorinating cultures enriched from Aarhus Bay marine sediments (24) were spiked three times with PCE at 250 µM (black arrows in Fig. 1B). The cultures were grown under sulfate-amended (S) and sulfate-free (NS) conditions, respectively. The NS_Tr2 and S_Tr2 cultures reductively dechlorinated PCE to cDCE, reaching a final concentration of 734.7 µM and 675.1 µM, respectively, after three spikes of 250 µM of PCE, with TCE as intermediate (Fig. 1B). The resulting biomass was collected for DNA isolation for metagenome sequencing after dehalogenation of the third spike of PCE (gray arrows in Fig. 1B). Similarly, the PCE_NS_Tr4 and PCE_S_Tr4 cultures reductively dechlorinated PCE to final concentrations of 636.8 µM and 528.8 µM cDCE, respectively, after three spikes of 250 µM of PCE. Biomass was collected when 80% of the third spike was dehalogenated to cDCE and was used for RNA extraction for metatranscriptome sequencing, thus increasing the chances of harvesting the OHR-associated genes that were active, especially the corresponding RDase genes. The cultures from the fourth transfer without sulfate fully dechlorinated the first spike of PCE within 14 days, whereas the full dechlorination of the first spike of PCE required 30–34 days in all other sulfate-amended cultures. This is probably due to competition between sulfate and PCE as the final electron acceptor, as was also observed in our previous study (24). The electron donor and carbon source, lactate, was consumed with the formation of propionate and acetate at a ratio of around 2.5:1 in cultures not amended with sulfate, whereas only acetate was produced in cultures where sulfate was added.

### Abundance and expression of bins in PCE dechlorinating cultures

**Fig 2:**
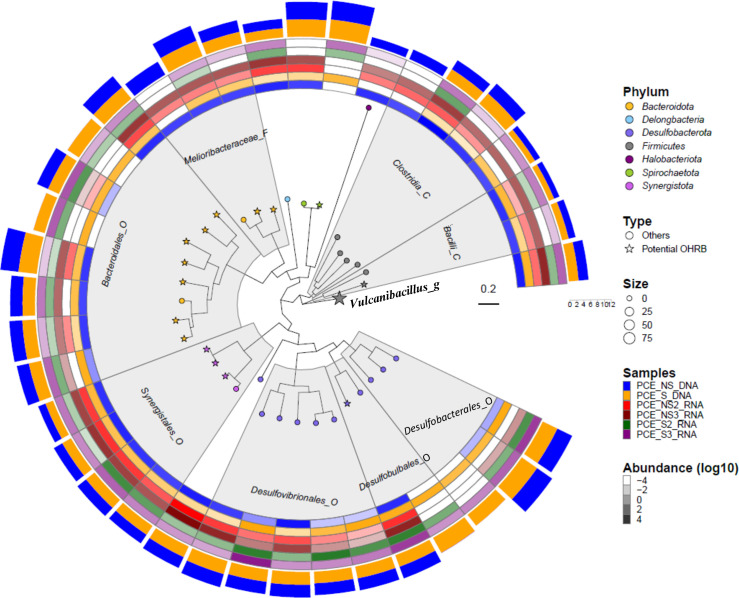
Phylogenomic tree of assembled bins constructed on the basis of 71 bacterial marker genes as implemented in Anvi’o. Tips of the tree labeled with the star symbol indicate bins containing RDase genes (“Potential OHRB”), with symbol size representing the number of RDase genes. Colors of tree tips indicate their classification at the phylum level. Areas shaded in gray indicate bins belonging to the same taxon at the level of order (_O), class (_C), or family (_F). The combined heatmap in the outer circles in different colors indicates the presence of bins sourced from metagenomic (PCE_DNA) and meta-transcriptomic (PCE_RNA) data from cultures grown in the absence or presence of sulfate (NS or S). Transparency of the colors in the heatmap represents the abundance of a given bin (Unit: genome copies per million reads) in log10. The two outer circles bearing the same color pattern as the metagenomic samples indicate the presence, and the bar height represents the bin’s genome size as measured by the scaler (0–12 Mbp).

Combining the DNA and RNA sequence data of both NS and S cultures, 37 assembled bins were retained based on completeness (>75%) and contamination (<5%) thresholds (Table S2). Four of these bins, bin.15 (classified into genus *Desulforhopalus*), bin.22 (classified into *Desulfobacter*), bin.34 (classified into *Pseudodesulfovibrio*), and bin.5 (classified into order *Synergistales*) had 100% completeness and no contamination according to the CheckM output (29, 60). The 37 assembled bins were classified into six bacterial phyla, *Bacteroidota*, *Delongbacteria*, *Desulfobacterota*, *Firmicutes*, *Spirochaetota*, and *Synergistota*, and one archaeal phylum, *Halobacteriota*, according to the taxonomic classification following the workflow of GTDB-Tk (classify_wf) (Fig. 2). The bins belonging to *Delongbacteria* and *Halobacteriota* did not encode any putative RDase genes, indicating that these organisms were probably not involved in the dechlorination of PCE.

In the NS cultures, bin.17, classified into *Clostridiaceae*, and bin.9, classified as a member of *Desulfobacterota*, were dominant on average with 12299.6 and 3401.2 genome copies per million reads (reflecting how often a given bin is represented in the sequence data), respectively (Table S2), followed by bin.26 classified as a member of *Vulcanibacillus* with 1831.6 genome copies. Bin.21, classified into *Desulfuromusa,* was the most highly expressed genome and accounted for 9101.4 genomic transcripts per million reads on average, followed by bin.15 classified as a member of *Desulforhopalus* with 673.9 genomic transcripts per million reads. The genomes of bin.14 (classified into *Desulfomicrobium*) and bin.26 were also highly represented in expression data with over 300 genomic transcripts per million reads.

When sulfate was present, bin.19, belonging to *Desulfoplanes*, was the most abundant and highly expressed bin, accounting for 6076.2 genome copies and 1145.1 genomic transcripts per million reads, followed by bin.22 with 3103.4 copies and 106.5 transcripts per million reads. Bin.34, belonging to *Pseudodesulfovibrio,* was the third most abundant with 3095.2 genome copies and 19.3 transcripts per million reads. Bin.25, belonging to the same genus, had 333.7 transcripts as the second most highly expressed bin with 666.8 genome copies per million reads (details shown in Table S2). Bin.26 belonging to the genus *Vulcanibacillus* was annotated with 97 putative RDase genes. Interestingly, under sulfate-free (NS) conditions, no transcript reads were detected that could be mapped to the genomes of bin.6 (*Bacteroidota*), bin.8 (*Desulfobacterota*), bin.23 (*Bacteroidota*), bin.27 (*Desulfobacterota*), and bin.30 (*Spirochaetota*), whereas reads mapped to bin.11 (*Firmicutes*), bin.29 (*Bacteroidota*), and bin.33 (*Halobacteriota*) were not detected under sulfate-amended (S) conditions. Other genomic bins containing RDase genes, bin.5, bin.10, and bin.32, belonging to *Synergistales*, bin.15 from *Desulfobacterota*, bin.28 belonging to *Melioribacteraceae*, and bin.12, bin.18, bin.24, and bin.31 from *Bacteroidales* were present and expressed in all cultures during PCE dechlorination irrespective of the presence of sulfate, whereas all their correspondent RDase genes were also expressed (Fig. 2; Table S5). In the sulfate-amended cultures, bin.23 and bin.6, belonging to the order *Bacteroidales*, and bin.30, belonging to the *Oceanispirochaeta*, became active alongside the sulfate reduction, as evidenced by the detection of transcripts that could be mapped to these genomes (Fig. 2; Tables S2 and S5). In addition, the potential OHRB, bin.15 belonging to *Desulforhopalus* and bin.32 belonging to *Synergistales*, were also found to be expressed under sulfate-added conditions, and their correspondent RDase gene transcripts were observed with an average of 65.3 (bin.15-RDase gene) and 145.5 (bin.32-RDase gene) transcripts per million reads, respectively (Table S5).

### Phylogenomic position of bins representing potential OHRB

**Fig 3:**
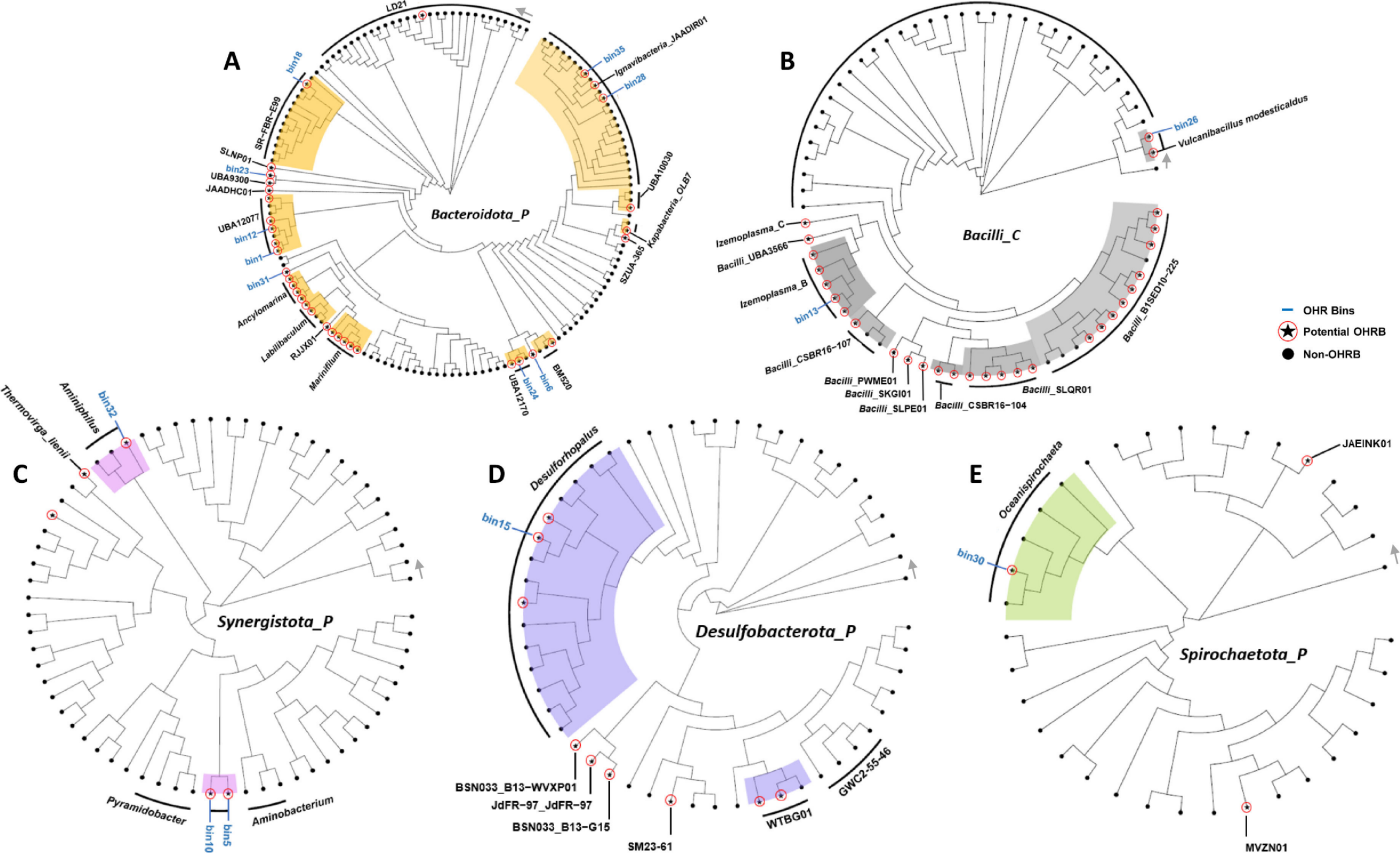
Phylogenomic trees of phyla including bins containing RDase genes. Representative genomes at phylum level: *Bacteroidota* (**A**), *Synergistota* (**C**), *Desulfobacterota* (**D**), and *Spirochaetota* (**E**) and class level *Bacilli* (**B**). Red circles filled with stars indicate potential OHRB, whereas black-solid dots represent non-OHRB. Colored shades indicate branches and nodes containing bins that encode RDase genes, in line with the color patterns used in Fig. 2 corresponding to the classification at phylum level *Bacteroidota* (orange), *Firmicutes* (gray), *Synergistota* (pink), *Desulfobacterota* (blue), and *Spirochaetota* (green). Gray arrows indicate the reference genomes, and bins are listed in an anti-clockwise order in Table S3.

To further assess the phylogeny of bins containing RDase genes and thus assumed to bear OHR potential and assess whether genomes of close relatives of the bins also code for this potential, representative genomes of the different taxa were retrieved from the GTDB database to construct phylogenomic trees. Nine OHR-potential bins, bin.1, bin.12, bin.18, bin.23, bin.24, bin.28, bin.6, bin.31, and bin.35, belonging to *Bacteroidota*, were phylogenetically analyzed with representative genomes (Fig. 3A). Bin.1 and bin.12 belong to genus UBA12077, in which three out of six representative genomes were annotated as potential OHRB. Bin.18 belongs to the genus SR-FBR-E99, and no RDase was found encoded in the genomes of any of the representatives. Bin.23 was classified into family VadinHA17, which included four potential OHRB representatives from genera UBA9300, JAADHC01, SLNP01, and LD21, of which UBA9300 was most closely related to bin.23. Bin.31 was phylogenetically close to *Marinifilaceae*, which contains genera *Ancylomarina*, *Marinifilum*, *Ancylomarina*_A, and *Labilibaculum*. Interestingly, we found RDase genes in *Ancylomarina*, *Marinifilum*, and *Ancylomarina*_A. Bin.28 and bin.35 from the family *Melioribacteraceae* were close to the potential OHRB family member, JAADIR01. Bin.24 was classified to genus UBA12170, which has in total two OHRB candidates across the included representatives. Bin.6 was in a close relationship with genus BM520, in which one of three representatives was noted as an OHRB candidate. Bin.13 was classified into genus *Izemoplasma*_B in the phylum *Firmicutes*, in which all five representative genomes were predicted to encode the potential for OHR (Fig. 3B). Bin.26 was affiliated with *Vulcanibacillus*, which has one isolate, *V. modesticaldus*, bearing one putative RDase gene. Bin.32, belonging to the *Synergistota*, was close to *Aminiphilus*. In addition, bin.5 and bin.10 were phylogenetically close to *Aminobacterium* (Fig. 3C). Within the *Desulfobacterota*, bin.15 was the closest to *Desulforhopalus* genomes that included two that were predicted to encode OHR potential among the ten representative genomes included in the analysis (Fig. 3D). Bin.30 from *Spirochaeota* was classified into *Oceanispirochaeta* (Fig. 3E).

### Genomic survey for PCE dechlorination concurrent with sulfate reduction and methanogenesis, respectively

**Fig 4:**
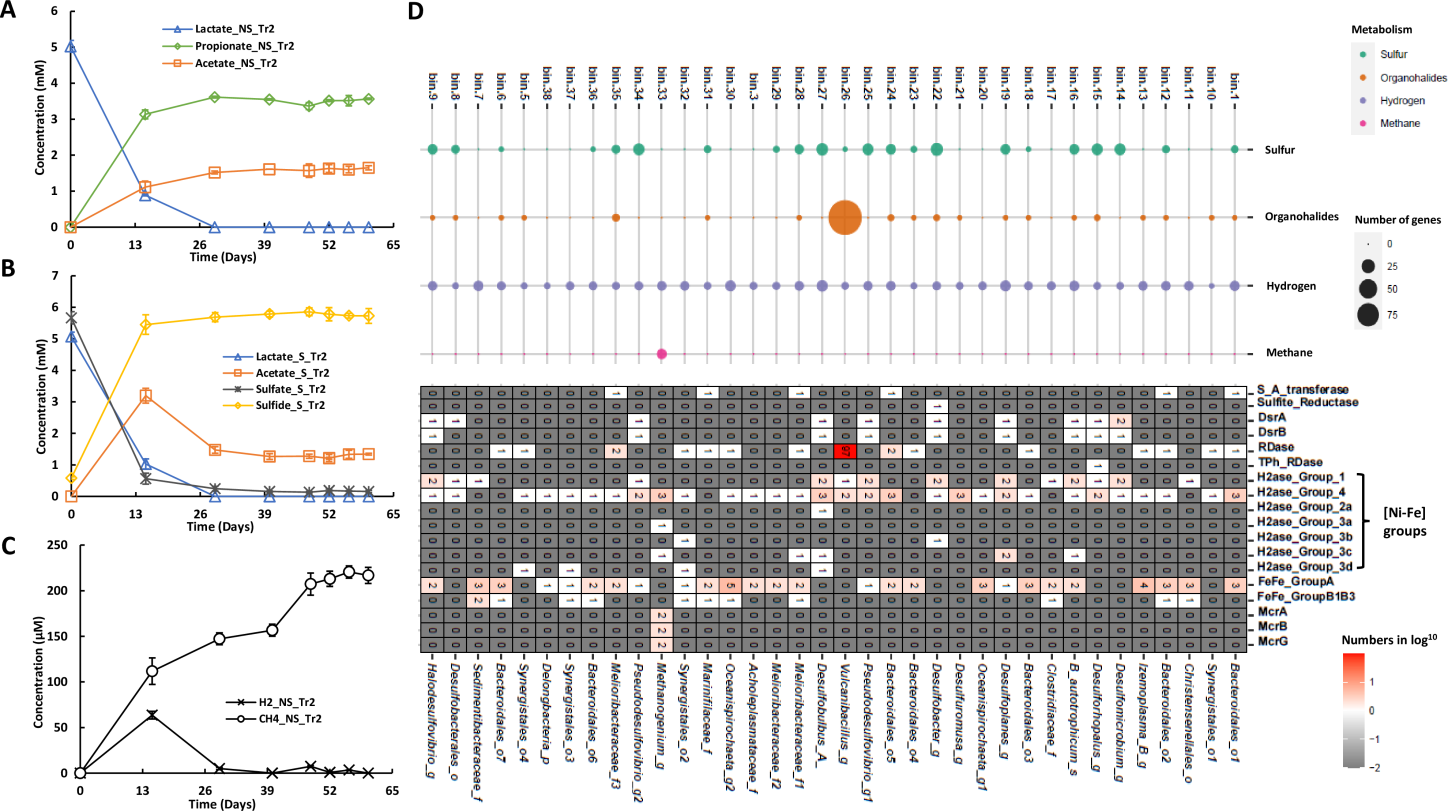
Metabolite detection and annotation of related marker genes. Utilization of lactate under sulfate-free, NS (**A**), and sulfate-amended, S (**B**), conditions. Hydrogen and methane were only detected in NS cultures (**C**); marker genes encoding proteins involved in the cycling of sulfur, halogens, hydrogen, and methane were searched against the bins and counted (**D**); thereupon, specific marker genes were selected corresponding to physiological observations (**D**). Bars in panels A, B, and C represent the standard errors of the duplicate cultures as described in Fig. 1. S_A_transferase, sulfate adenylyl-transferase; Sulfite_reductase, sulfite reductase; DsrA, dissimilatory sulfite reductase alpha subunit; DsrB, dissimilatory sulfite reductase beta subunit; RDase, reductive dehalogenase; TPh_RDase, tetrachloro-P-hydroquinone RDase; H2ase, hydrogenase; FeFe-GroupA and FeFe-GroupB1B3, [Fe-Fe]-hydrogenases in Group A and Group B1B3, respectively; McrA, McrB, McrG, alpha, beta, and gamma subunits of Methyl-coenzyme M reductases, respectively.

Twenty-two bins were found encoding genes coding for proteins involved in sulfate reduction, of which bin.1, bin.12, bin.24, bin.28, bin.31, and bin.35 contained genes encoding sulfate adenylyl-transferase (S_A_transferase) that initiates the reduction of sulfate to sulfite with adenosine 5’-phosphosulfate (APS) as the intermediate. Bin.14, bin.15, bin.16, bin.19, bin.22, bin.25, bin.27, bin.34, bin.8, and bin.9 were annotated with sulfite reductases catalyzing the further reduction of sulfite to sulfide (Fig. 4D). Interestingly, there was no assembled bin bearing the complete gene set for sulfate reduction to sulfide.

We identified 15 bins containing genes predicted to code for respiratory RDases and one TPh-RDase from bin.15 belonging to *Desulforhopalus*. Hydrogen was produced during lactate utilization and PCE dechlorination up to around 70 µM and consumed again after 26 days. Meanwhile, methane was also detected and accumulated to around 240 µM in the absence of added sulfate (Fig. 4C). Probably, the produced hydrogen provided the electrons for reductive dehalogenation and methanogenesis under these conditions. Further genomic analysis revealed that marker genes encoding group A [Fe-Fe]-hydrogenases were abundant across the bins, for example, bin.30 and bin.13 carried five and four genes, respectively, and several bins were also found to encode subgroup 1 and 4 types [Ni-Fe]-hydrogenases (Fig. 4D). The higher numbers found of genes coding for [Fe-Fe]-hydrogenase suggested that they were likely the main contributors to hydrogen oxidation in accordance with previous results that [Fe-Fe]-hydrogenases were more active and had higher turnover frequency than [Ni-Fe]-hydrogenases (61). In addition, bin.23, bin.31, and bin.6 only contained genes encoding [Fe-Fe]-hydrogenases, whereas bin.10, bin.14, bin.15, bin.21, and bin.26 only contained genes encoding [Ni-Fe]-hydrogenases. Genes coding for methyl-coenzyme M reductases (*mcr*) contributing to methane production were only found in bin.33, classified into *Methanogenium*, which contained two genes of each *mcrA*, *mcrB*, and *mcrG*.

### Phylogeny of bin-associated RDases

**Fig 5:**
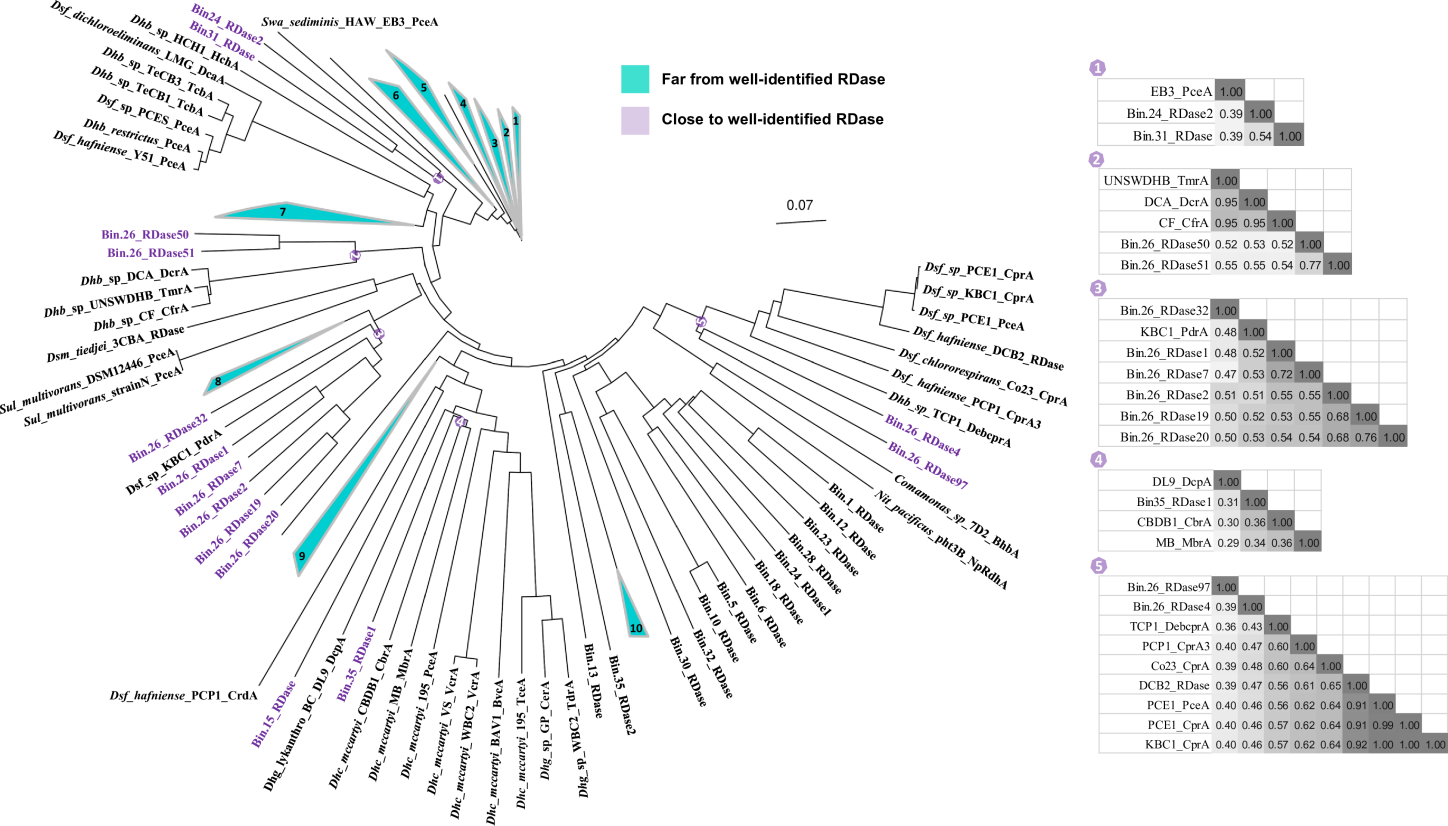
Phylogenetic analysis of bins’ RDases including well-identified RDase representatives. RDases in bin.26 distinct to the well-identified RDase representatives were divided into 10 groups in turquoise. The number of genes from group 1 to group 10 is 3, 7, 13, 7, 10, 12, 20, 4, 5, and 3, respectively. In contrast, branches containing RDases from bins as well as well-identified RDase representatives at the same node were numbered and shaded in light purple from left to right. For each shade, full-length protein pairwise identity (PID) matrices are given in the right panel. In the matrices, the gray color intensity is proportional to the PID. Columns from left to right are in the same order as rows from top to bottom. The scale bar of 0.07 represents the evolutionary distance, meaning 7 substitutions per 100 amino acid sites on average.

Most RDase genes in bin.26 (*Vulcanibacillus*) were found clustered in one assembled contig, with less than five gene intervals between every two RDase genes (see gene locus numbers in Tables S4 and S5). Most RDases from bin.26 were phylogenetically closely related to each other with respect to their amino acid sequences (Fig. 5; Table S4). RDases from other bins, except bin.35, bin.15, bin.31, and bin.24, were in a close phylogenetic relationship to each other but distant to the RDases from bin.26 (Fig. 5), suggesting a different origin. Furthermore, RDases from the assembled bins were distinct from the established OGs shown in Fig. 5 (36, 37). Despite their low full-length PID to members of OGs (< 60%), some RDases shared >35% PID with OGs. Among these were seven RDases from bin.26 that were phylogenetically more closely related and shared over 50% PID with known RDases, including RDase50 and RDase51 bearing, respectively, 52.9% and 55.5% PID to DcrA from *Dehalobacter* sp. strain DCA (62), and RDase1, 2, 7, 19, and 20 sharing, respectively, 52.4%, 50.8%, 52.6%, 52.5%, and 53.1% PID with PdrA from *Desulfitobacterium* sp. strain KBC1 (39). In addition, three other RDases from bin.26 exhibited a phylogenetic relationship with well-characterized proteins, including RDase32 (48.4%) to PdrA from *Desulfitobacterium* sp. strain KBC1 (39), RDase4 (47.6%) to CprA from *Desulfitobacterium chlororespirans* strain Co23 (41), and RDase97 (40%) to CprA from *Desulfitobacterium* sp. strain KBC1 (39). Three RDases from other bins also clustered with known RDases, including RDase2 from bin.24 and the RDase from bin.31 bearing both 38.7% identity to PceA from *Shewanella sediminis* strain HAW-EB3 (38), and RDase1 from bin.35 having 35.7% PID to CbrA from *Dehalococcoides mccartyi* strain CBDB1 (63).

### Expression of RDase genes during PCE dechlorination

**Fig 6:**
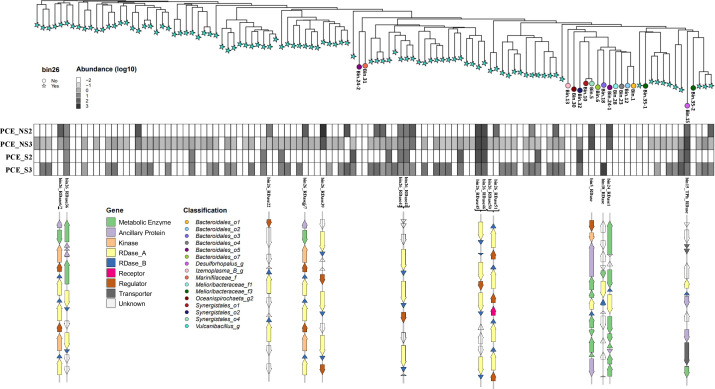
Phylogenetic tree of RDases and their gene transcripts attached with corresponding gene clusters. The protein sequences of RDases were collected and aligned via online Clustal Omega. Stars at the nodes indicated that RDases were encoded on Bin.26. RDases encoded on other bins were depicted by differently color-filled circles, and RDases in the same bin were in the same color. The extended heatmap corresponding to the RDase nodes indicated gene transcript abundance (log10-transformed) for two pairs of duplicate cultures including PCE_NS2, PCE_NS3, PCE_S2, and PCE_S3. Gray color intensity in the heat map is proportional to the transcript abundance. The depicted gene clusters represent RDase genes that are transcribed in both duplicates of NS and S, only in duplicates of NS, or only in S. RDase41/42, RDase45/46, and RDase6/7 are in the same cluster, respectively (Table S5). The neighborhood genes encoding diverse proteins related to RDase genes on the clusters were distinguished by distinct colors. Operons were drawn using gggenes and adjusted individually to fit the figure size. Therefore, a uniform scale bar for gene length was not applied.

RDase gene expression profiles were analyzed to specify their possible contributions to PCE dechlorination. No RDase gene transcripts were observed for bin.1, bin.6, bin.10, bin.12, bin.13, bin.23, bin.24, bin.28, bin.30, bin.31, or bin.35 (Fig. 6). Moreover, no transcripts of bin.6, bin.23, or bin.30 were detected under sulfate-free conditions (Table S2). A total of 24 RDase genes were not expressed under any of the conditions tested in this study, half of which were from bin.26 (Table S5). Hence, it is unlikely that those genes were involved in the dechlorination of PCE. In contrast, transcripts of several RDase genes of bin.26 were observed regardless of the presence of sulfate, including RDase3, RDase9, RDase21, RDase23, RDase43, RDase54, RDase58, RDase60, RDase61, RDase64, RDase75, RDase79, and RDase96. Thirty-one RDase genes were only expressed in NS cultures, of which 29 RDase genes were from bin.26. Furthermore, 23 RDase genes from bin.26 were only found expressed in PCE_NS3. Only one RDase gene of bin.18 was transcribed in PCE_S3 culture with 508.2 transcripts (Table S5). All the other RDase genes were expressed to different extents with and without sulfate. The RDase genes from bin.15 and RDase36, RDase41, RDase42, RDase45, and RDase46 from bin.26 were expressed in all duplicate cultures (Fig. 6). Notably, 84 of the 97 RDase genes from bin.26 were found to be expressed during PCE dechlorination, which suggests the important role of bin.26 to dehalogenate PCE in the cultures described here. A total of 39 RDase genes were expressed over 20 transcripts under either NS or S conditions. These included 34 RDase genes in bin.26, one in bin.5, bin.15, and bin.18, respectively, and RDase1 gene in bin.24. Furthermore, three pairs of RDase genes in high expression, including RDase6 and RDase7, RDase41 and RDase42, and RDase45 and RDase46, were not only physically connected in tandem on the genome but also shared the same number of transcripts (Fig. 6). Combining the conserved motifs and expression files of these respiratory RDases, we found that all the putative RDases retain two conserved binding motifs for Fe-S clusters, except for RDase72 from bin.26, which lacks the second Fe-S binding motif, Fe-S2, as well as the TAT motif. Nine of 88 expressed RDase genes from bin.5, bin.18, bin.24 (RDase1), bin.32, and bin.26 (RDase33, RDase82, RDase83, RDase84, and RDase97) under NS and S conditions have incomplete or missing TAT motifs (Fig. S2; Table S5), suggesting that the RDase activity could be executed intracellularly. The genes neighboring the RDase gene were diverse (Fig. 6), with transcriptional regulator-encoding genes being frequently detected, followed by genes coding for metabolic enzymes and associated cofactors, such as cyclases, hydrolases, and chaperone proteins.

### Diverse regulatory systems flanking RDase genes

**Fig 7:**
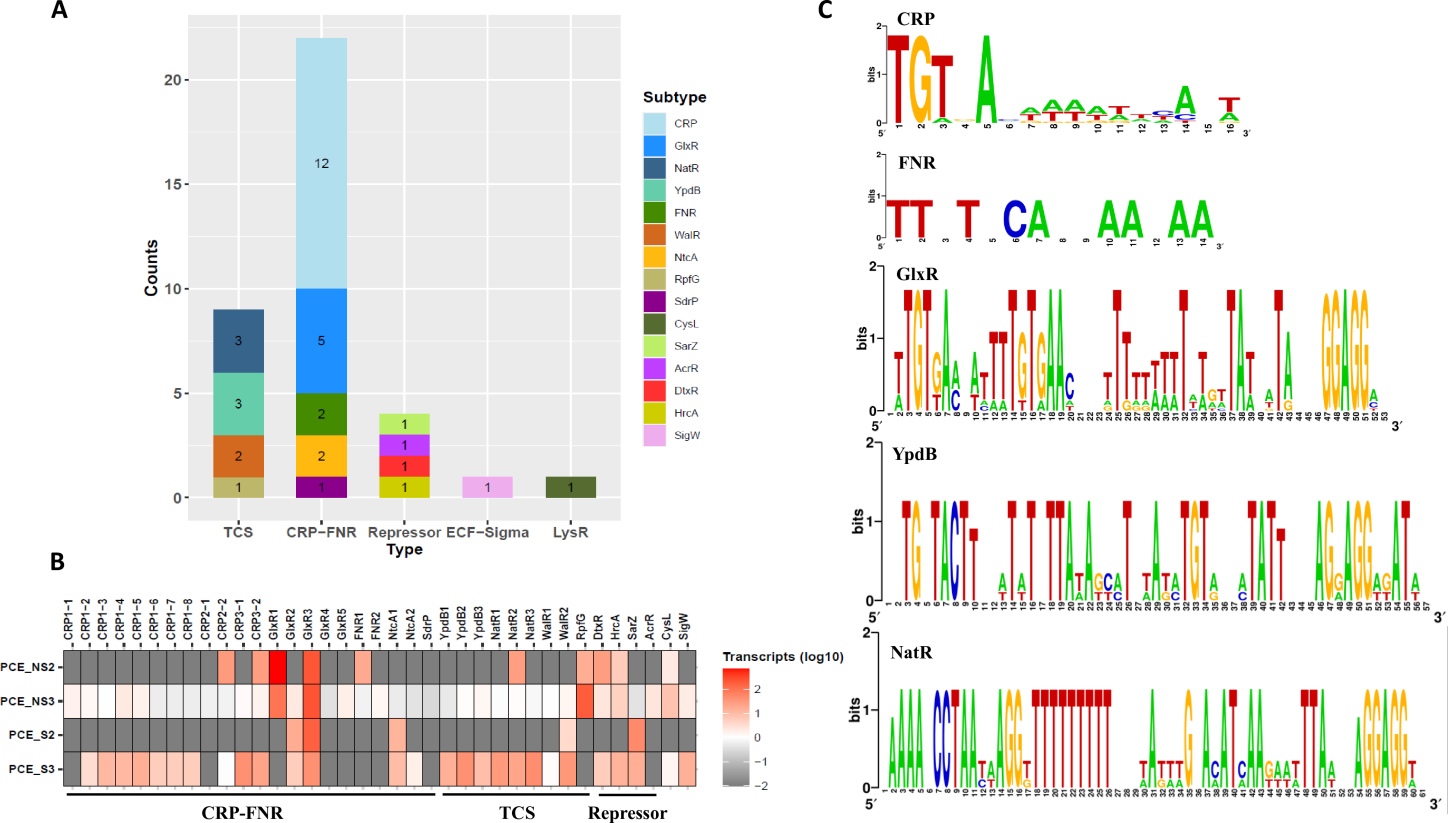
Classification and transcript abundance of expressed transcriptional regulators (**A, B**) and their predicted binding motifs (**C**). **(A**) Groups of transcriptional regulators, TCS, two-component systems; CRP-FNR, CRP (cyclic AMP receptor protein)/FNR (fumarate and nitrate reduction regulator); ECF-Sigma (ECF-σ): Extra-cytoplasmic function sigma factor; PCE_NS2 and PCE_NS3: duplicate PCE dechlorinating cultures without additional sulfate; PCE_S2 and PCE_S3: duplicate PCE dechlorinating cultures with additional sulfate; the potential binding sites were identified on the basis of binding motif in the potential promoter regions and displayed in Weblogo (https://weblogo.berkeley.edu/logo.cgi). The y-axis depicts the degree of conservation (in bits) for each position in a sequence alignment.

Genes coding for transcriptional regulators adjacent to RDases were subjected to further analysis. Genes encoding CRP/FNR and TCS were the main expressed transcriptional regulator genes, including 22 (5 subtypes) and 9 (4 subtypes) genes, respectively (Fig. 7A). CRP, the cAMP-activated global transcriptional regulator, was represented by 12 members, divided further into three groups based on sequence similarities (64). CRP-2 and CRP-3 were represented by a higher number of transcripts compared with CRP-1 (Fig. 7B). Five members of GlxR (65), a CRP-like regulator, were found expressed, with GlxR1-3 exhibiting higher transcription levels compared with the rest.

Nine two-component systems (TCS) were tentatively identified based on their identity to known systems; among them, NatR2 was previously found to be involved in regulating sodium ion extrusion (66), whereas WalR2, shown to be involved in cell wall metabolism and virulence, and RpfG, involved in signaling response, were highly expressed (Fig. 7B). Interestingly, four transcriptional regulators previously found to function as repressors were also highly expressed, including DtxR shown to inhibit Diphtheria toxin production, HrcA involved in preventing heat-shock induction, SarZ for attenuating virulence, and AcrR shown to be involved in global stress response (67–70). In addition, CysL, belonging to LysR-type regulators related to L-Cysteine biosynthesis (71), and SigW, an extra-cytoplasmic function (ECF) sigma factor responding to alkaline shock stress (72), were also induced during PCE dechlorination (Fig. 7B). To better understand the regulons of these expressed transcriptional regulators, we obtained promoter sequences of RDase genes by defining intergenic regions shorter than 300 bp between the RDase gene and the upstream gene (Table S6). There were 12 promoter sequences collected, and each was found to contain binding sites following the consensus for CRP regulons of *E. coli* (5′-TGTGACAAAATTCA*T-3′, MX000093: CRP, PRODORIC) (73) (Fig. 7C). In a similar way, the predicted binding sites of FNR followed the consensus 5′-TT*T*CA**AA**AA-3′ according to the two promoters of RDase genes following the consensus for known FNR regulons (MX000004: Fnr, PRODORIC). The binding sites of GlxR, YpdB, and NatR were predicted based on their RDase genes’ promoter alignment. Two promoter candidate sequences, associated with RDase4 and RDase70 of Bin.26, were assumed to be under the regulation of NtcA (MX000209: NtcA), of which only the promoter sequence of RDase70 was found to contain the binding site, 5′-GTATAAATATAAAC-3′.

## DISCUSSION

Our previous work demonstrated that marine sediments from Aarhus Bay can dehalogenate a broad range of organohalides, including PCE, under sulfate-amended and sulfate-free conditions (24), which for the first time provided physiological evidence of OHR for marine sediments of Aarhus Bay (18–20, 74, 75). Therefore, further pinpointing OHRB populations residing in Aarhus Bay marine sediments was the logical next step. To this end, we applied metagenomics and metatranscriptomics, yielding 37 assembled bins with high quality, of which 15 bins were predicted to represent OHRB due to their genomic annotation with respiratory RDase genes. The taxonomic classification of these OHR bins unveiled a wide distribution of RDase genes among members of *Bacteroidota*, *Snergistota*, and *Spirochaetota* in addition to phyla well-known to include OHRB, such as *Firmicutes* and *Desulfobacterota* (76). Moreover, agreeing to the previous study (24), the presence of sulfate shaped the PCE-dechlorinating microbial community significantly with respect to MAG diversity and their relative abundance (Table S2). Evidently, the inclusion of sulfate in the medium inhibited methanogenesis (bin.33, belonging to genus *Methanogenium*), which was in line with the physiological observation (Fig. 4C) and previous studies due to the competitive advantage of sulfate reducers for substrates, that is, hydrogen (24, 77). Similarly, competition for hydrogen by sulfate reducers could prevent the growth of obligate OHRB. Noticeably, putative OHRB bins, including bin.6, bin.23, and bin.30, were not detected under sulfate-free conditions, and for none of these bins, RDase gene transcripts were detected under any of the conditions tested (Table S5), implying their RDase genes were less likely responsible for PCE dechlorination. Therefore, experimental settings with various organohalides could provide a wider scenario of dehalogenators expanding to the new phyla in addition to the well-documented counterparts in Aarhus Bay marine sediments under S and NS conditions. Gene expression analyses indicated it is likely that one or more of the RDases encoded on bin.26, bin.15, bin.24, bin.18, and bin.5 played the main role in PCE dechlorination, in which bin.26 was phylogenetically close to *Vulcanibacillus modesticaldus*, and bore an unprecedentedly high number of 97 different RDase genes, which is significantly more than the copy numbers in typical OHRB (e.g., *Dehalococcoides mccartyi* CG1 with 36 RDase genes [36]). In addition, most of the induced RDase genes were clustered with genes encoding diverse regulatory systems, suggesting their strong flexibility in adapting to environmental changes or the availability of different organohalides.

### Wide distribution of RDases beyond well-characterized OHRB

Most of the OHRB have so far been isolated from organohalide-contaminated areas, such as soils, rivers, and lakes (78–80). They belong to the phyla *Chloroflexota*, *Firmicutes*, *Desulfobacterota*, and *Proteobacteria* (76, 81). Although pristine marine sediments have been shown to contain OHRB such as *Dehalococcoides* (82), fewer OHRB were isolated from pristine marine environments, which might be due to the limited knowledge of their metabolic potential and limited methods for isolation. The integration of metagenomics and metatranscriptomics employed in this study allowed for the assembly of 15 putative OHR bins with high quality, some of which belong to *Bacteroidota*, *Spirochaetota*, and *Synergistota* phyla that have never been reported to catalyze reductive dehalogenation. The average nucleotide identity (ANI) of these OHR bins was below 95% in all cases after running the “classify_wf” of GTDB, indicating that they likely represent different species. Our further exploration together with representative genomes of these phyla revealed that 112 genomes of *Bacteroidota*, 15 genomes of *Spirochaetota*, and seven genomes of *Synergistota* are bearing RDase genes, with none of the corresponding isolates being physiologically characterized to perform OHR. To this end, it is noteworthy that our study using genome-resolved strategies discovered that the RDase genes from bin.18, classified into *Bacteroidota*, and from bin.5 and bin.32, classified into *Synergistota*, were expressed in PCE dechlorinating cultures (Table S2), and their genome abundances and genomic transcripts were in high numbers, suggesting that OHR potential exists in these phyla. The RDase genes in the other bins from the above phyla were not expressed in our data, indicating that they were probably not involved in the dehalogenation of PCE in our experiments. We found 32 representative genomes from the class *Bacilli* that are annotated with RDase genes, and bin.26 is closely related to *Vulcanibacillus,* which has one isolate from a moderately hydrothermal vent containing one RDase gene (83). In contrast, bin.26 was predicted to contain 97 RDase genes, a number that is higher than the maximum number of 36 currently reported for members of *Dehalococcoides* (36, 84, 85). Interestingly, these RDases were divergent in protein sequences and showed low pairwise identity (PID) with the classified ortholog groups (36, 37), suggesting that they were novel groups to the well-identified RDases, especially for PCE dechlorination. In addition, most of the RDases in the other OHR bins also showed relatively low PID with known RDases, indicating their distinct origination and the fact that they could target different organohalides (Fig. 5). Furthermore, more detailed inspection of bin.26 using tools geared at the detection of virus-associated sequences (VIBRANT and VirSorter2 [86–88]) revealed the presence of prophage fragments (data not shown). Accordingly, it is tempting to speculate that the RDase genes from bin.26 could be the result of horizontal gene transfer. *Desulfobacterota* is a phylum reclassified from Deltaproteobacteria and well known for catalyzing sulfate reduction (81). Eighty-nine of the 939 representative genomes in the *Desulfobacterota* were found to carry RDase genes, similar to previously described strains of *Desulfoluna*, *Desulfuromusa*, and *Desulfovibrio* (25, 89). In contrast, bin.21, classified into *Desulfuromusa*, had no annotated genes involved in OHR or sulfate reduction and had a high abundance when sulfate was absent, as was also found in the preceding study (24). Interestingly, bin.21 was predicted to encode three group 4 [Ni-Fe]-hydrogenase genes and could thus act as a potential hydrogen producer in our cultures. Noticeably, the higher numbers found of genes coding for [Fe-Fe]-hydrogenase suggested that they were likely the main contributors to hydrogen production in accordance with previous results that [Fe-Fe]-hydrogenases were more active and had higher turnover frequency than [Ni-Fe]-hydrogenases (61). One bin, bin.15, was most closely affiliated with the genus *Desulforhopalus*, of which one putative OHRB is *D. singaporensis* (90). Noticeably, the deduced RDase from bin.15 does not belong to the canonical RDases associated with OHR, such as PceA from *Dehalococcoides* (91, 92), and was more similar in sequence and size to TPh-RDase from *Sphingobium chlorophenolicum* (previously named as *Flavobacterium* sp.) that can dehalogenate tetrachlorohydroquinone thiolytically (6, 7). Of interest, the TPh-RDase from bin.15 was not reported for respiratory dehalogenation to conserve energy; however, the transcript data revealed that TPh-RDase bears potential biodegrading activity of PCE in the cultures studied here. Moreover, the genomic abundance and transcript of bin.15 indicated its participation in PCE dechlorination. Thus, further metabolic analysis of bin.15 is essential. Altogether, the marine sediments of Aarhus Bay are home to several new OHRB candidates with diverse RDase genes.

### RDase gene clusters

Recently, an extensive genomic survey found that some RDase gene clusters lack genes encoding anchor protein B or the N-terminus of RDase bears transmembrane domains, which could leave the RDase functioning in the cytoplasm or the C-terminus spanning toward the outer face of the membrane (93, 94). In our study, we also observed some OHR bins without putative RdhB encoding genes, including bin.12, bin.1, bin.28, bin.32, and bin.6. Of these, transcripts of the RDase gene in bin.32 indicated possible cytoplasmic dehalogenation that awaits further experimental confirmation. In most cases, RDase genes were accompanied by diverse functional gene sets, such as cobamide cofactor biosynthesis pathway genes in *Sulfurospirillum* strains and molecular chaperones in *Desulfitobacterium* (95–97). Similarly, some RDase gene clusters found in the present study contained chaperone genes vicinal to genes encoding RDase3, RDase4, RDase35, and RDase93 of bin.26 that could protect the RDase activity in a manner of maintaining structural integrity when exposed to harsh environments. Besides, there were several genes encoding electron transport complexes accompanying RDase genes, including RDase in bin.13 and bin.31, RDase2 in bin.24 and bin.35, and RDase4, RDase67, RDase69, RDase73, RDase82, and RDase84 in bin.26, which could form new electron transport chains to promote OHR in addition to the previously identified ones (3, 98).

Furthermore, RDase genes were frequently flanked by genes coding for transcriptional regulators, which might timely and accurately regulate the expression of vicinal RDase genes in response to the added organohalides. Three regulatory systems were previously characterized in association with RDases, including those of the CRP-FNR family, MarR and TCS (8, 9, 96, 97, 99). Expressed transcriptional regulators classified into CRP/FNR systems accounted for the largest numbers in our study, whereas, GlxR, as a new regulator, showed different regulons inferred from the promoter alignment as well as YpdB and NatR from the class of TCS. In addition, four negative regulators were found that could follow the modulation reported for MarR-type regulators (9), but their binding sites were unclear and needed further demonstration. Noticeably, ECF sigma factor, SigW, was also found transcribed during PCE dechlorination, which was rarely reported to regulate dehalogenation, and its DNA binding motif seems different from that in *Bacillus subtilis* (MX000079: SigW). Taken together, the newly discovered regulatory systems diversified the known modes of regulation of dehalogenation that could be the result of adaptation to challenging environments, and further molecular identification would substantially improve our understanding of reductive dehalogenation and its regulation in natural systems.

## Data Availability

The nucleotide sequence data have been deposited at the European Bioinformatics Institute under accession number PRJEB87757.

## SUPPLEMENTAL MATERIAL

Figure S1 msystems.01503-24-s0001.tiff Workflow of metagenomic analysis.

Figure S2, part 1 msystems.01503-24-s0002.tiff Protein sequence alignment of the putative reductive dehalogenases.

Figure S2, part 2 msystems.01503-24-s0003.tiff Protein sequence alignment of the putative reductive dehalogenases.

Legends msystems.01503-24-s0004.docx Legends for supplemental figures and tables.

Supplemental tables msystems.01503-24-s0005.xlsx Tables S1 to S6.

ASM does not own the copyrights to Supplemental Material that may be linked to, or accessed through, an article. The authors have granted ASM a non-exclusive, world-wide license to publish the Supplemental Material files. Please contact the corresponding author directly for reuse.
