# Supplementary figures and images for: Genome-resolved transcriptomics reveals novel PCE-dehalogenating bacteria from Aarhus Bay sediments
Source: mSystems. 2025 Apr 16;10(5):e01503-24. doi: 10.1128/msystems.01503-24 (PMC12090745; doi:10.1128/msystems.01503-24)

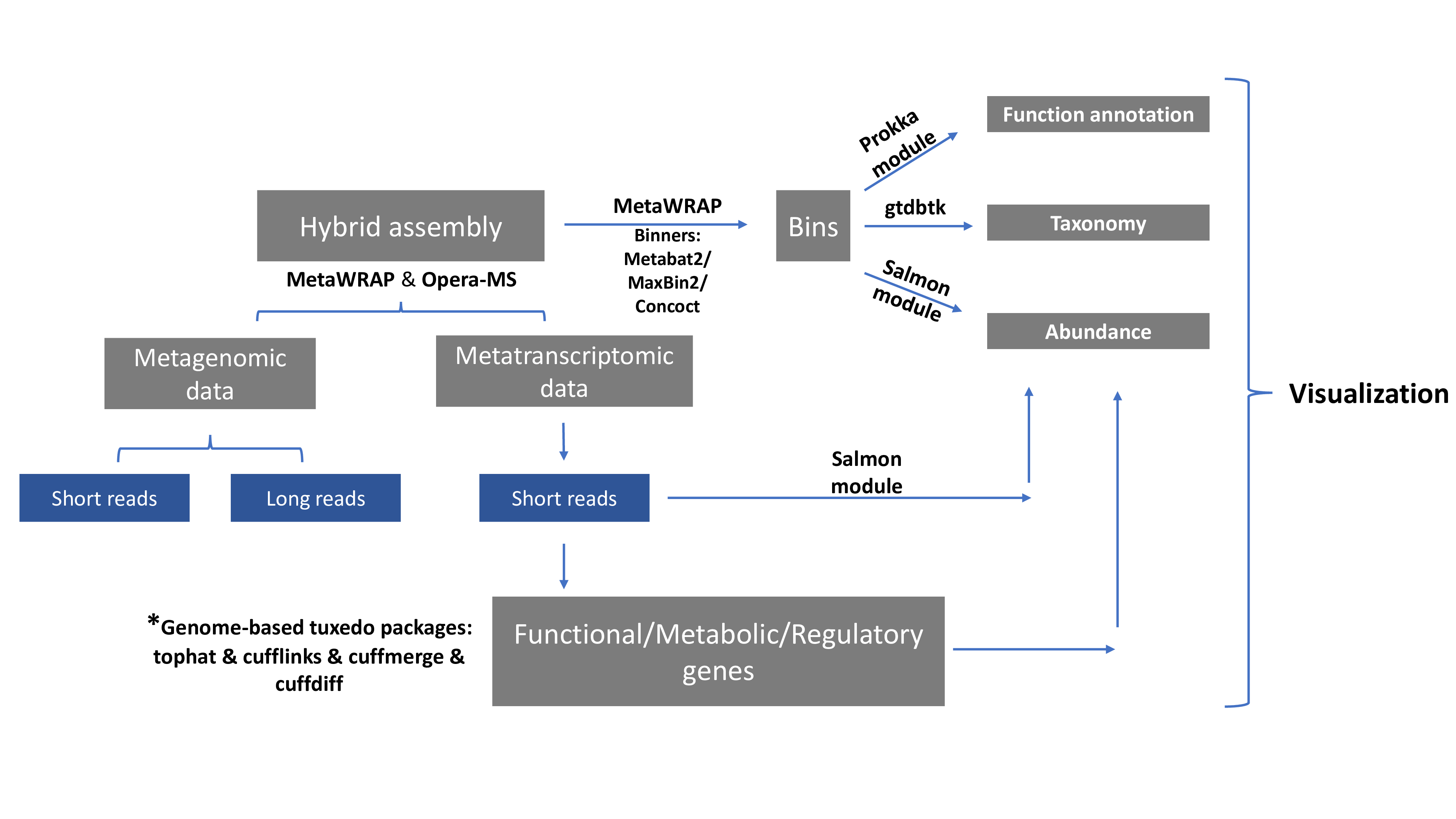

Supplement: Figure S1 — Workflow of metagenomic analysis. [file msystems.01503-24-s0001.tiff]

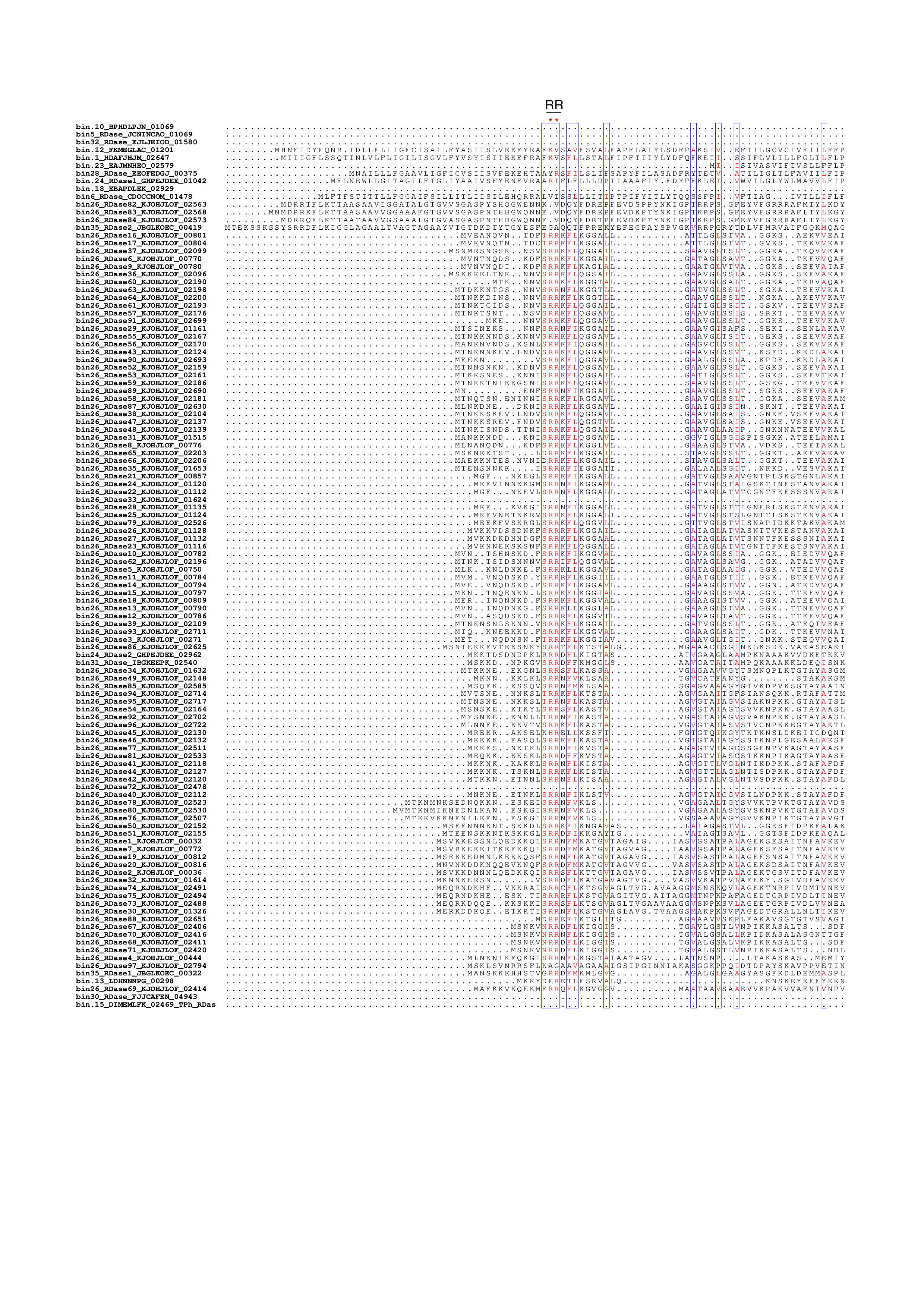

Supplement: Figure S2, part 1 — Protein sequence alignment of the putative reductive dehalogenases. [file msystems.01503-24-s0002.tiff]

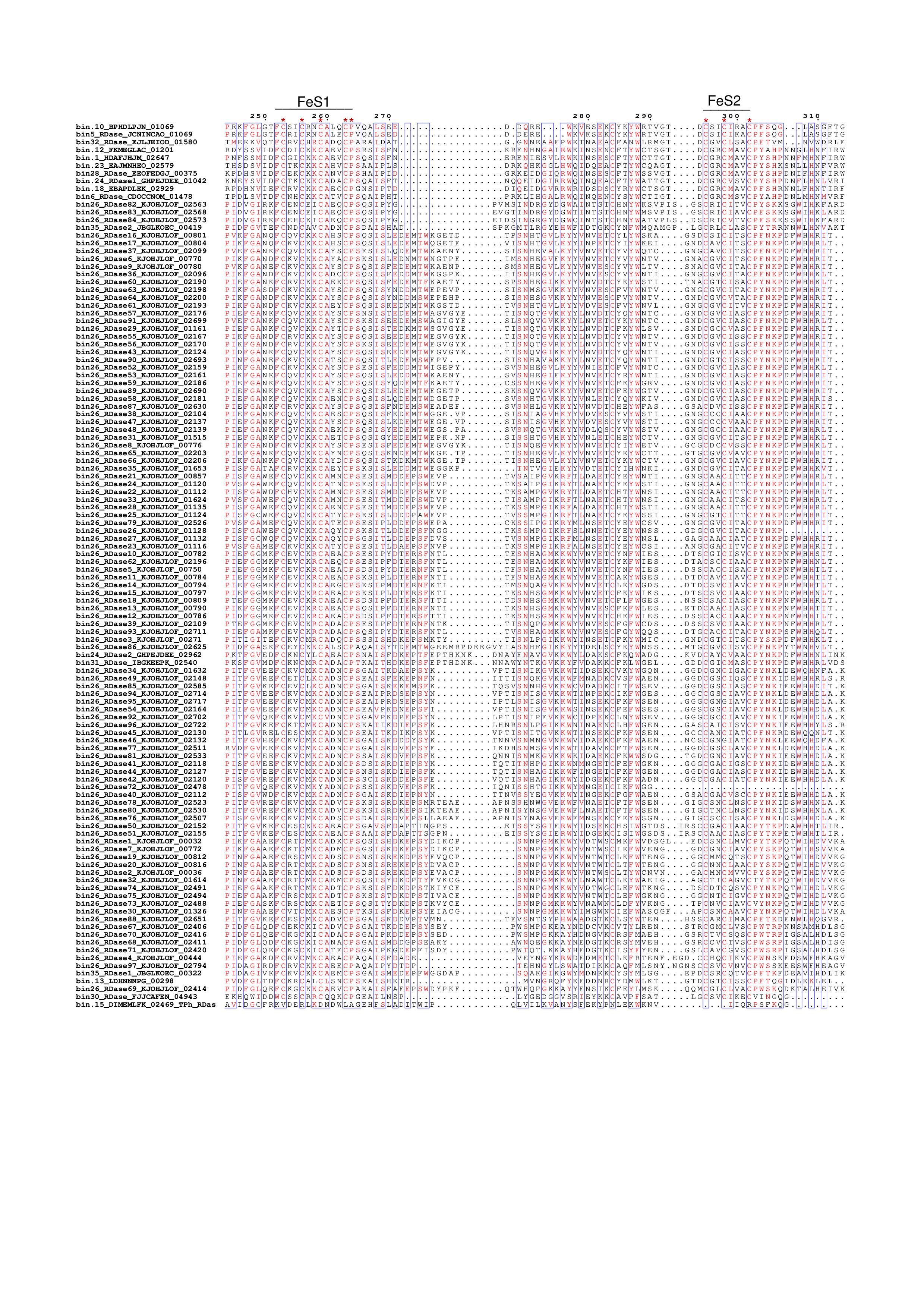

Supplement: Figure S2, part 2 — Protein sequence alignment of the putative reductive dehalogenases. [file msystems.01503-24-s0003.tiff]
