# Supplementary material for: Genome-resolved transcriptomics reveals novel PCE-dehalogenating bacteria from Aarhus Bay sediments
Source: mSystems. 2025 Apr 16;10(5):e01503-24. doi: 10.1128/msystems.01503-24 (PMC12090745; doi:10.1128/msystems.01503-24)
Supplement: Legends — for supplemental figures and tables. [file msystems.01503-24-s0004.docx]

**Supplementary**

**Genome-resolved transcriptomics reveals novel PCE-dehalogenating bacteria from Aarhus Bay sediments**

Chen Zhang^a,b☒^, Tom N.P. Bosma^a,c^, Siavash Atashgahi^a,d^, Hauke Smidt^a☒^

^a^ Laboratory of Microbiology, Wageningen University & Research, Stippeneng 4, 6708 WE Wageningen, The Netherlands

^b^ Current address: Wenzhou Institute, University of Chinese Academy of Sciences, Wenzhou 325000, China

^c^ Current address: Deltares, Daltonlaan 600, 3484 BK, Utrecht, The Netherlands

^d^ Current address: AB Mauri, Oude Kerkstraat 55, 4878 AK , Etten-Leur, The Netherlands

^☒^Corresponding author: Laboratory of Microbiology, Wageningen University & Research, Stippeneng 4, 6708 WE Wageningen, The Netherlands. Tel: +86 18810891307; E-mail: [chen03.zhang@gmail.com](mailto:chen03.zhang@gmail.com);

Laboratory of Microbiology, Wageningen University & Research, Stippeneng 4, 6708 WE Wageningen, The Netherlands. Tel: +31317483102; E-mail: [hauke.smidt@wur.nl](mailto:hauke.smidt@wur.nl);

**Figure Legends**

**Figure S1.** Outline of metagenomic and meta-transcriptomic sequence processing and analyses. * Analyses of meta-transcriptomic data was performed by tuxedo packages that include tophat (aligning reads), cufflinks (assembling transcripts), cuffmerge (merging transcripts) and cuffdiff (identifying differentially-expressed transcripts) (1). Arrows in blue indicate stepwise analyses. Visualization of output data was achieved using R and the required packages, including ggplot2, ggtree, and gggenes.

**Figure S2.** Multiple sequence alignment of putative RDases from assembled bins. * Indicates the conserved sites of RDases motifs, twin-arginine (RR) translocation signal peptide at N terminus (page 1), and two binding motifs of iron-sulfur clusters, FeS1 and FeS2, at C terminus (page 2). Multiple sequence alignment was executed by online Clustal Omega tool and visualised by ESPript (<https://espript.ibcp.fr/ESPript/ESPript/>).

**Supplementary tables**

**Table S1** Metagenomic assembly, using short and long read DNA sequences as well as short read metatranscriptome sequences.

**Table S2** Metagenome Assembled Genomes (MAGs) with abundance of genomic copies and transcripts in per million reads unit.

**Table S3** Phylogenomically-close representatives to RDase-containing bins.

**Table S4** Protein sequences of RDases in assembled bins.

**Table S5** Transcriptional profile of RDase genes in assembled bins.

**Table S6** Predicted promoter sequences of RDase genes.

**Reference**

1. Trapnell C, Roberts A, Goff L, Pertea G, Kim D, Kelley DR, Pimentel H, Salzberg SL, Rinn JL, Pachter L. 2012. Differential gene and transcript expression analysis of RNA-seq experiments with TopHat and Cufflinks. *Nat Protoc* 7:562-578.
